# Infectious Triggers and Immune Dynamics in Guillain–Barré Syndrome: Revisiting *Campylobacter jejuni* and the Silent Role of *Haemophilus influenzae*

**DOI:** 10.1002/mbo3.70177

**Published:** 2025-11-27

**Authors:** Aswathi Ramesh, Rajasekaran Subbarayan, Dhasarathdev Srinivasan, Ranjith Balakrishnan, Rupendra Shrestha, Ankush Chauhan

**Affiliations:** ^1^ Centre for Advanced Biotherapeutics and Regenerative Medicine, Faculty of Research, Chettinad Hospital and Research Institute, Chettinad Academy of Research and Education Kelambakkam India; ^2^ Centre for Herbal Pharmacology and Environmental Sustainability, Chettinad Hospital and Research Institute, Chettinad Academy of Research and Education Kelambakkam India; ^3^ Department of Natural and Applied Sciences Nexus Institute of Research and Innovation (NIRI) Lalitpur Nepal

**Keywords:** *Campylobacter jejuni*, Guillain–Barré Syndrome, *Haemophilus influenzae*, immune dysregulation, neuropathy

## Abstract

Guillain–Barré Syndrome (GBS) is a rapidly progressing immune‐mediated neuropathy that remains the leading cause of acute flaccid paralysis worldwide. A substantial proportion of GBS cases are precipitated by infectious agents, most notably *Campylobacter jejuni* and *Haemophilus influenzae*, which initiate pathogenic autoimmunity via molecular mimicry. This narrative review aimed to synthesize current evidence on the microbial triggers of GBS and elucidate the immune mechanisms linking infection to neuropathic damage. We discuss the evolving landscape of GBS pathogenesis, emphasizing the roles of ganglioside‐like lipooligosaccharide (LOS), host genetic predisposition, and dysregulated immune responses. The clinical heterogeneity of GBS subtypes, including axonal and demyelinating variants, was analyzed in relation to serotype‐specific antibody profiles that inform the diagnosis and prognosis. Current therapeutic interventions, including intravenous immunoglobulin and plasma exchange, are critically assessed alongside experimental strategies, such as monoclonal antibody therapies, microbiome modulation, and LOS‐targeted vaccines. This review highlights microbial surveillance and precision immunotherapy in the management of GBS. Collectively, this study underscores the central role of microbiological insights in redefining the prevention, diagnosis, and treatment of this complex neuroimmune disorder.

## Graphical abstract

Schematic representation of the gut‐brain axis and microbial triggers in Guillain–Barré Syndrome. Post‐infectious immune responses, particularly through molecular mimicry by pathogens, such as *Campylobacter jejuni* and *Haemophilus influenzae*, lead to demyelination and subsequent neuromuscular paralysis.

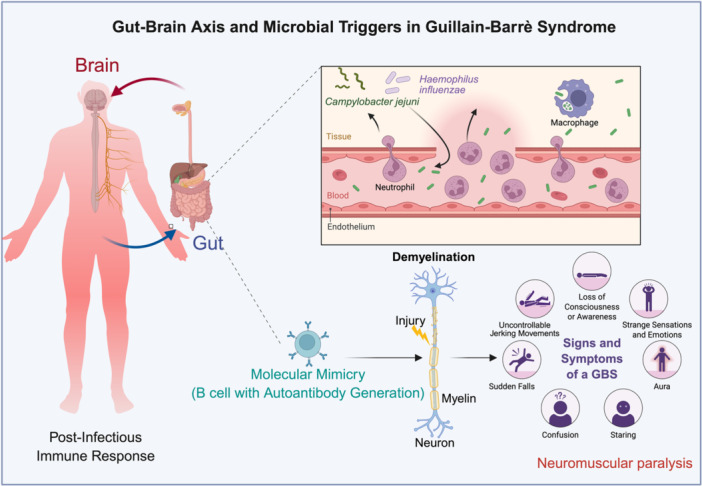

## 1 Introduction

Guillain–Barré Syndrome (GBS) is an acute immune‐mediated polyneuropathy that presents with rapidly progressing muscle weakness and paralysis in severe cases (Finsterer 2022). It is the leading cause of acute flaccid paralysis worldwide, surpassing poliomyelitis in terms of its incidence and clinical burden. GBS presents a significant clinical challenge owing to its unpredictable course and potential for long‐term disability (Elendu et al. 2024). The global annual incidence of GBS ranges between 0.8 and 1.9 cases per 100,000 individuals, increasing to approximately 2.7 per 100,000 among adults aged ≥ 80 years, with a slight male predominance (Bragazzi et al. 2021). Seasonal variations have been observed, likely reflecting fluctuations in the antecedent infections. Notably, certain regions report higher incidence rates, potentially linked to increased exposure to specific infectious agents and environmental factors (Willison et al. 2016). The substantial morbidity associated with GBS combined with its socioeconomic impact from prolonged hospitalization and rehabilitation underscores the need for a deeper understanding of its underlying mechanisms.

GBS is predominantly a post‐infectious disorder, with approximately two‐thirds of patients reporting symptoms of respiratory or gastrointestinal infections within 4 weeks before onset. Several pathogens have been implicated as triggers for this disease, among which *Campylobacter jejuni (C*. *jejuni)* is the most significant. Identified in 25–50% of GBS cases, *C. jejuni* infection is strongly associated with an acute motor axonal neuropathy (AMAN) variant, characterized by rapid progression and severe weakness (Nyati and Nyati 2013). The pathogenic mechanism involves molecular mimicry between *C. jejuni* LOS and human gangliosides, triggering an autoimmune response that damages the peripheral nerves (Stein 2022). Other infectious triggers, including cytomegalovirus (CMV), Epstein‐Barr virus (EBV), influenza A virus, and more recently, Zika virus, have also been implicated (Pinto‐Díaz et al. 2017). The emergence of arbovirus‐related GBS cases underscores the need for vigilant epidemiological monitoring (Αρμπουνιώτη 2024). In rare cases, *Haemophilus influenzae (H. influenzae)* has been identified as a potential bacterial trigger predominantly linked to the acute inflammatory demyelinating polyneuropathy (AIDP) subtype. Although rare, certain vaccinations have been temporally associated with GBS incidence. For instance, during the 1976 H1N1 influenza vaccination campaign, an increased incidence of GBS was observed, estimated at one additional case per 100,000 vaccinations (Wise et al. 2012). Subsequent studies have reported a much lower risk, emphasizing that the benefits of immunization outweigh potential risks. Collectively, these findings indicate that immune dysregulation following infection, rather than direct microbial invasion, underlies GBS pathogenesis.

The pathogenesis of GBS is complex and involves both humoral and cellular immune responses. Molecular Mimicry, the structural similarity between microbial antigens and peripheral nerve components, leads to the production of cross‐reactive antibodies. In *C. jejuni*, LOS structures mimic human gangliosides, resulting in the generation of antibodies that target nerve tissues, causing demyelination or axonal degeneration. Immune Response Modulation beyond humoral immunity and cell‐mediated responses, including the activation of T‐helper 17 cells and the release of pro‐inflammatory cytokines such as interleukin‐17 (IL‐17), contribute to nerve injury (Soltani et al. 2019). The exact interplay between these immune components remains an active area of research (Mishu and Blaser 1993). Microbiological research has been instrumental in elucidating these mechanisms of action. Advancements in molecular techniques have enabled the identification of specific microbial triggers and characterization of pathogenic pathways, paving the way for targeted therapeutic interventions. GBS typically presents with symmetrical limb weakness, areflexia, and varying degrees of sensory disturbances. AIDP is the most common form in Europe and North America and is characterized by demyelination of peripheral nerves (Alessandro et al. 2018). Another variant of GBS is AMAN, which is more prevalent in East Asia and is often associated with *C. jejuni* infection, leading to axonal degeneration without significant demyelination (Shang et al. 2021). Miller‐Fisher syndrome (MFS), a variant characterized by ophthalmoplegia, ataxia, and areflexia, is frequently associated with anti‐GQ1b antibodies. Diagnosis is primarily clinical, supported by electrophysiological studies and cerebrospinal fluid analysis, which show albuminocytological dissociation. Serological tests can detect recent infections, aiding in the identification of potential triggers (Mishu and Blaser 1993). The management of GBS focuses on supportive care and immunomodulatory therapy. Monitoring respiratory function is crucial, as approximately 20%–30% of patients may require mechanical ventilation. Multidisciplinary care, including physiotherapy and occupational therapy, is essential for rehabilitation (Vitacca et al. 2014). Plasma exchange (plasmapheresis) and intravenous immunoglobulin (IVIg) are the mainstay treatments that hasten recovery. The choice between these therapies depends on their availability, patient factors, and resource considerations. Corticosteroids alone have not been shown to be effective in altering the disease course. Ongoing research into the immunopathogenesis of GBS may lead to novel therapeutic targets, such as cytokine inhibitors or monoclonal antibodies, offering hope for more effective future treatments (Sprenger‐Svačina et al. 2024). This narrative review aimed to synthesize current evidence on the infectious triggers of GBS, particularly *C. jejuni* and *H. influenzae*, and to elucidate how microbial virulence, immune dysregulation, and host genetics converge to influence disease onset, clinical course, and therapeutic outcomes.

## 2 *C. jejuni* as a Principal Etiological Factor in Guillain–Barré Syndrome

### 2.1 Microbiological Profile and Pathogenic Mechanisms of *C. jejuni*

*C. jejuni* is a Gram‐negative, spiral‐shaped bacterium recognized as a leading cause of bacterial gastroenteritis globally (Liu 2021). Human infections typically arise from the ingestion of undercooked poultry, unpasteurized milk, contaminated water, or contact with infected animals, including domestic pets and livestock. Epidemiological data suggest that in addition to foodborne sources, recreational water exposure, such as swimming in untreated lakes and rivers, may contribute to sporadic outbreaks, although this is not consistently observed across all settings (DeFlorio‐Barker et al. 2018). Although gastroenteritis caused by *C. jejuni* is often self‐limiting, its ability to trigger a systemic immune response that leads to neurological damage raises significant concerns (Smith 1995). The neurovirulence potential of the bacterium is strongly influenced by strain‐specific genetic variations within its LOS biosynthesis loci, which govern molecular mimicry with host gangliosides (y Pérez 2018). The pathogenic arsenal of *C. jejuni* includes multiple virulence factors that facilitate its colonization and immune evasion. Its flagellar system not only mediates motility but also aids host cell adhesion and invasion (Kemper and Hensel 2023). Adhesins mediate adherence to intestinal epithelial cells, a critical step for colonization and subsequent invasion. Cytolethal Distending Toxin (CDT) induces DNA damage in host cells, leading to cell cycle arrest and apoptosis and contributing to tissue damage and inflammation (Jinadasa et al. 2011). These combined factors enable *C. jejuni* to breach host defenses and initiate immune responses capable of cross‐reacting with peripheral nerve components, thereby setting the stage for post‐infectious neuropathy.

### 2.2 Molecular Mimicry and Autoimmune Sequelae

**Figure 1:**
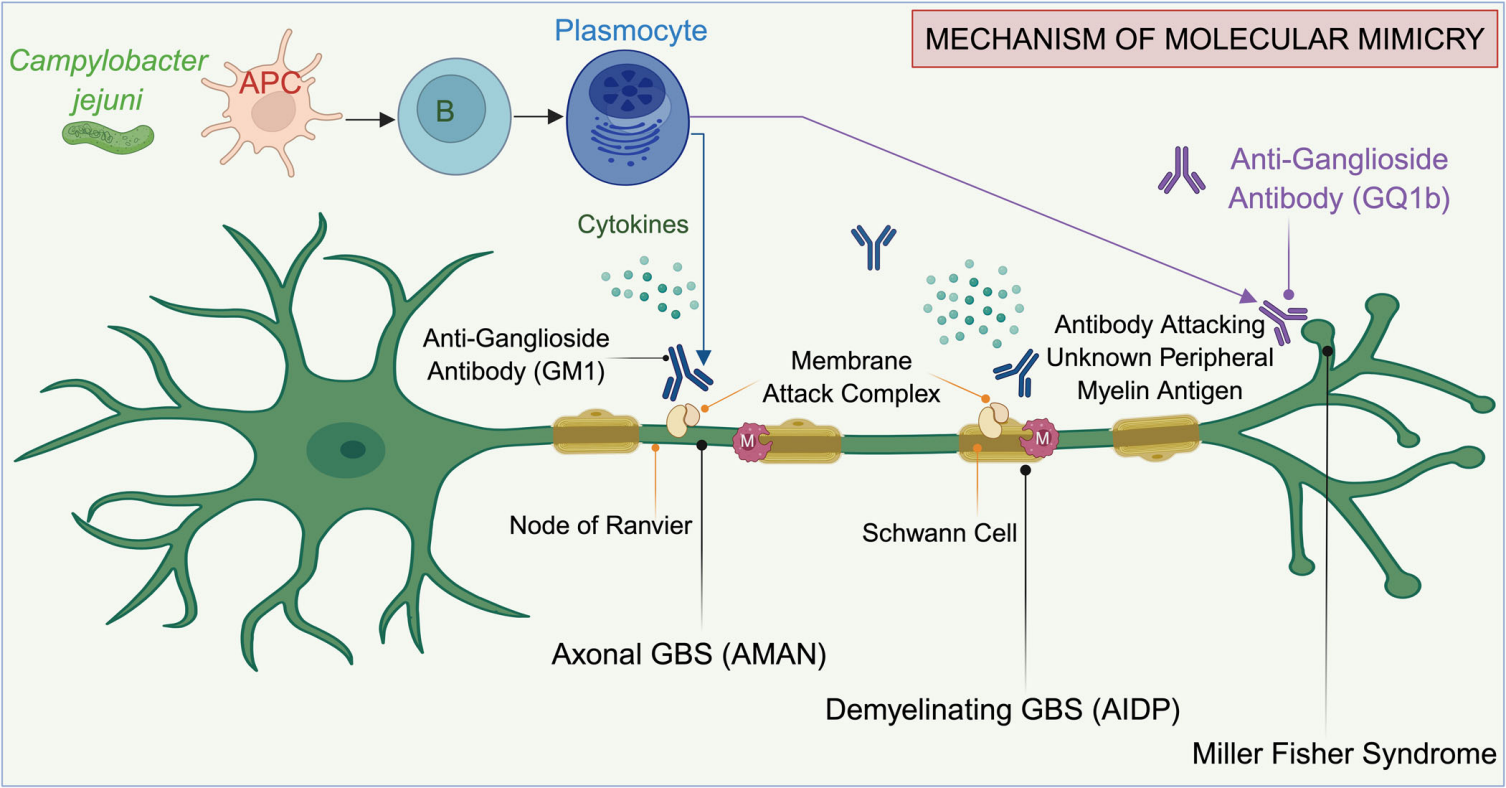
Illustration of *Campylobacter jejuni* infection triggering GBS through molecular mimicry. The immune system generates anti‐ganglioside antibodies (e.g., GM1 and GQ1b) that mistakenly attack the components of peripheral nerves, leading to axonal damage (AMAN), demyelination (AIDP), or Miller‐Fisher syndrome. APC, antigen‐presenting cells; B lymphocytes (B cells); GM1, monosialotetrahexosylganglioside; GQ1b, disialosylganglioside; AMAN, acute motor axonal neuropathy; AIDP, acute inflammatory demyelinating polyneuropathy; GBS, Guillain–Barré syndrome.

A defining feature of *C. jejuni*‐associated GBS is molecular mimicry, in which bacterial LOS structures resemble human gangliosides such as GM1, GD1a, and GQ1b (Figure 1). This antigenic similarity provokes the production of cross‐reactive antibodies that target both bacterial and host nerve membrane components, leading to immune‐mediated demyelination or axonal degeneration (Posse de Chaves and Sipione 2010). Notably, strains expressing the Cst‐II sialyltransferase gene produce highly sialylated LOS structures that enhance mimicry and increase the likelihood of neuropathic outcomes (Suliman 2024). The LOS biosynthesis loci in *C. jejuni* are highly variable, comprising multiple classes (A–E) that differ in sialytion genes and glycosyltransferases, thereby influencing the antigenic mimicry of gangliosides (Hameed et al. 2020). Frequent recombination within these loci generates strain diversity that shapes the neuropathogenic potential. Such genetic mosaicism explains the geographical variations in GBS subtypes, with certain LOS classes (notably A and B) correlating with the AMAN variants in East Asia. In contrast, non‐sialylated LOS types are more often associated with AIDP presentations in Europe and North America (Chiu et al. 2022; Yu et al. 2012; Dingle et al. 2005). The presence of anti‐GM1 and anti‐GD1a antibodies is strongly associated with the AMAN variant of GBS, whereas anti‐GQ1b antibodies are characteristic of Miller Fisher syndrome (MFS) (Oldstone 2006). Host susceptibility appears to modulate the course of autoimmune disease. Genetic polymorphisms in HLA‐DQB1 and HLA‐DRB1 alleles as well as variations in immune regulatory genes have been linked to an increased risk of molecular mimicry‐induced neuropathy (Kaida et al. 2009). In addition, recent findings indicate that gut microbiota dysbiosis following *C. jejuni* infection may amplify immune activation, enhance autoantibody production, and worsen nerve injury (Chiba et al. 1993). Collectively, these mechanisms underscore the intricate host‐pathogen interplay that determines disease expression and severity in GBS.

### 2.3 Epidemiological Correlations and Clinical Implications

Epidemiological studies indicate that 30%–40% of GBS cases are preceded by *C. jejuni* enteritis (McCarthy and Giesecke 2001). The risk of developing GBS varies geographically, reflecting differences in strain distribution, host genetics, and sanitation standards (Tikhomirova et al. 2024). Geographic and seasonal variations are well recognized, with higher incidences observed in warmer months, coinciding with increased poultry consumption and outdoor activities (Thépault et al. 2020). In addition to poultry, domestic pets, and cattle, contaminated recreational water has been identified as an occasional reservoir for neurovirulent strains. Although *C. jejuni*‐related BGS represents a small proportion of total infections, its clinical manifestations tend to be more severe, often involving axonal degeneration and prolonged recovery. Research efforts toward vaccine development against *C. jejuni* have gained momentum in recent years. Experimental approaches include conjugate vaccines targeting LOS and subunit vaccines using flagellin or capsule components to elicit protective immunity without inducing autoimmunity (Xu and Moyle 2017). However, the development of a safe vaccine remains challenging because of the risk of molecular mimicry with human gangliosides. Such preventive strategies hold promise for reducing the GBS incidence associated with infection, particularly in endemic regions.

## 3 *H. influenzae* and Its Role in Guillain–Barré Syndrome: A Neglected but Emerging Association

### 3.1 Microbiological Characteristics and Virulence Determinants

**Figure 2:**
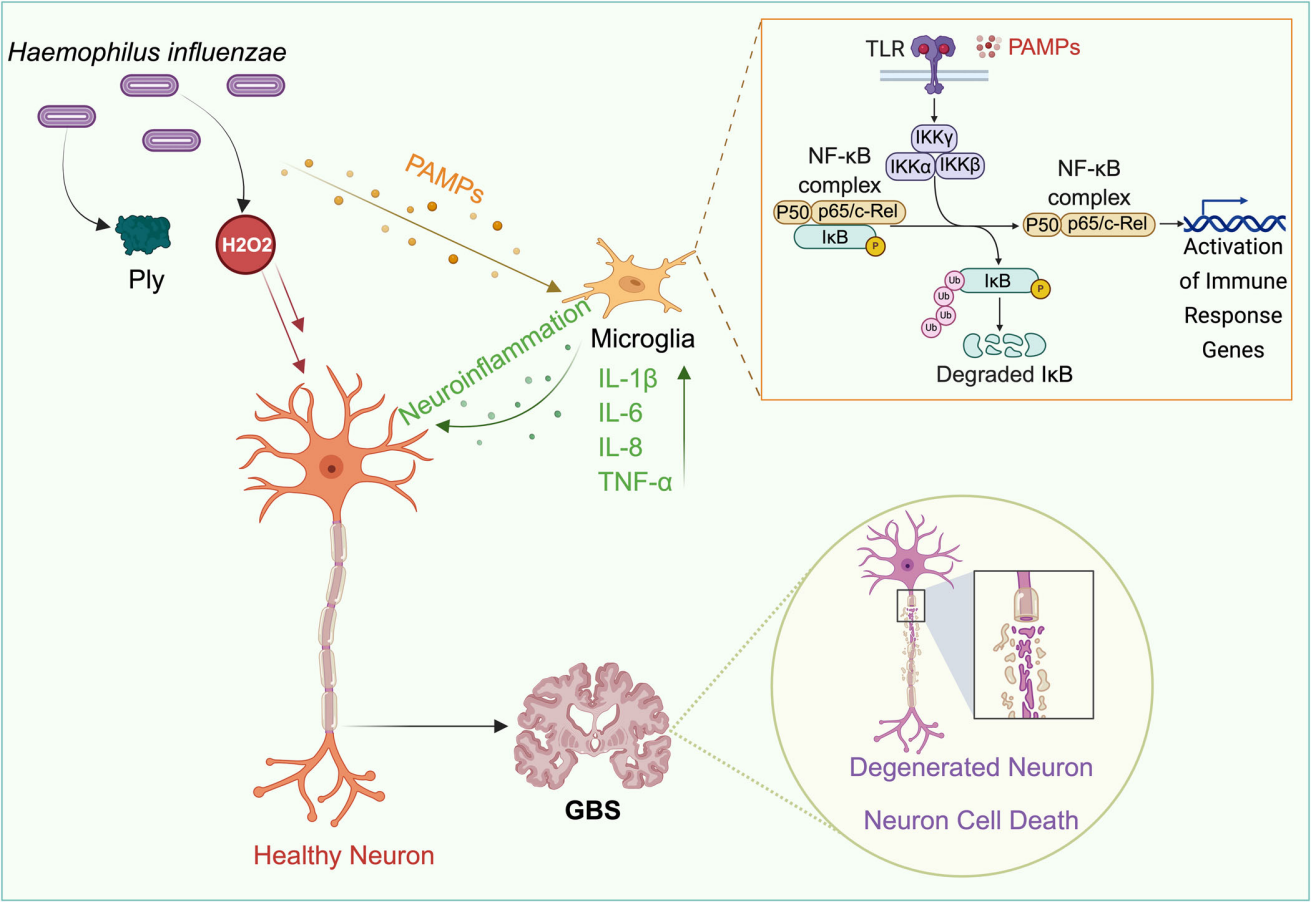
Illustration of the hypothetical mechanism by which *Haemophilus influenzae* may cause GBS by releasing toxins (such as H_2_O_2_ and Ply) that damage neurons and activate microglia. Microglia respond to bacterial PAMPs by triggering the NF‐k pathway and releasing inflammatory cytokines (IL‐1, IL‐6, IL‐8, and TNF‐α). This results in neuroinflammation, axonal degeneration, and neuronal death, which are hallmarks of GBS. Ply, pneumolysin; H₂O₂, hydrogen peroxide; PAMPs, pathogen‐associated molecular patterns; TLR, toll‐like receptor; IKK, IκB kinase; IKKα, IκB kinase alpha; IKKβ, IκB kinase beta; IKKγ, IκB kinase gamma; IκB, inhibitor of κB; NF‐κB, nuclear factor kappa‐light‐chain‐enhancer of activated B cells; IL‐1β, interleukin‐1 beta; IL‐6, interleukin‐6; IL‐8, interleukin‐8; TNF‐α, tumor necrosis factor‐alpha; GBS, Guillain–Barré syndrome.

*H. influenzae* is a facultative anaerobic, Gram‐negative coccobacillus traditionally recognized for its role in the respiratory tract, otitis media, and meningitis, particularly in children (High 2002). Increasing evidence suggests that *H. influenzae*, especially non‐typeable strains, may also act as potential triggers of autoimmune neuropathies, including Guillain–Barré Syndrome (Dalakas 2015). Although this association remains less well‐established than that of *C. jejuni*, the pathogen exhibits several virulence traits that could plausibly mediate post‐infectious immune cross‐reactivity (Figure 2). Although *H. influenzae* infections are common globally, their contribution to GBS has remained largely underrecognized, hence the term “silent role”. This stems from the low frequency of confirmed cases, limited serological screening for *H. influenzae*‐specific antibodies, and overlapping clinical features of *C. jejuni*‐associated neuropathies. Epidemiological data indicate that *H. influenza*‐associated GBS accounts for less than 5% of all post‐infectious cases, with sporadic reports across Europe, Japan, and North America often following upper respiratory tract infections (Finsterer 2022).

*H. influenzae* occurs in both encapsulated (typable) and nonencapsulated (non‐typable) forms, each displaying distinct mechanisms of pathogenicity. Encapsulated strains, particularly serotype b (*Hib*), exhibit robust polysaccharide capsules that enable resistance to complement‐mediated killing and phagocytosis (Vinuesa et al. 2001). In contrast, non‐typable *H. influenzae* (NTHi) strains depend on a diverse array of outer membrane proteins (OMPs) and LOS for adherence, immune evasion, and persistence within mucosal tissues (Osman et al. 2018). In addition, NTHi adhesins, including HMW1/HMW2 and Hap, mediate strong epithelial binding and contribute to chronic colonization (Osman et al. 2018). The LOS of *H. influenzae* shares partial structural homology with human gangliosides, raising the possibility of molecular mimicry as a mechanism underlying postinfectious autoimmunity. Strain‐specific differences in LOS sialylation may further influence its immunogenicity and neuropathogenic potential (Day et al. 2012). Additionally, toxins, such as hydrogen peroxide (H_2_O_2_) and Ply (pneumolysin‐like) proteins, have been proposed to contribute to neuronal injury and microglial activation (Armbruster 2011). Collectively, these virulence attributes underscore the potential of *H. influenzae* as a neuropathogenic cofactor, warranting greater attention in GBS research.

### 3.2 Immune Dysregulation and Molecular Mimicry: Parallels With *C. jejuni*

The immunopathogenesis of *H. influenzae*‐associated GBS likely parallels that of *C. jejuni* but may involve distinct immune pathways. While *C. jejuni* predominantly induces anti‐ganglioside antibodies(anti‐GM1 and anti‐GD1a), *H. influenzae* appears to elicit a broader spectrum of immune responses, including the production of autoantibodies against myelin‐associated glycoproteins (MAGs) and Schwann cell surface proteins (Sangster et al. 2019). These antibodies have been detected in patients with AIDP‐type neuropathy, suggesting a different immunological target spectrum (Mihajloska et al. 2021).

Infection with *H. influenzae* can trigger polyclonal B cell activation and upregulation of Toll‐like receptor 4 (TLR4) signalling via LOS recognition, leading to elevated production of pro‐inflammatory cytokines, such as IL‐1β, IL‐6, IL‐8, and TNF‐α (Tavares et al. 2017). This inflammatory milieu amplifies autoreactive responses and may account for the predominance of the demyelinating (AIDP) subtype observed in reported cases. Additionally, host factors such as specific HLA haplotypes (e.g., HLA‐DQB1 and DRB1) and gut microbiome composition likely modulate susceptibility, mirroring patterns observed in *C. jejuni*‐mediated GBS. results from molecular mimicry combined with host immune predisposition; however, definitive mechanistic evidence remains limited (Brooks 2016).

### 3.3 Clinical and Experimental Corroboration: Establishing Causality

Clinical documentation linking *H. influenzae* infection to GBS remains sparse, with fewer than a dozen published case reports and small case series describing this association to date (Mahammed 2023). These reports typically involve AIDP presentations following respiratory infections, especially in pediatric and immunocompromised individuals. Experimental studies provide partial mechanistic support; immunization of animal models with *H. influenzae* LOS has been shown to induce autoantibody production and peripheral nerve dysfunction reminiscent of GBS (Nachamkin et al. 2008). In vitro assays have similarly demonstrated that exposure to *H. influenza*‐derived antigens can trigger Schwann cell injury and complement activation (Berger et al. 2013). However, systematic epidemiological data and large‐scale immunological investigations are lacking.

Given these limitations, interpretations of *H. influenzae‐associated* GBS causality should remain hypothesis‐driven rather than conclusive. The limited case data suggest a plausible association but not yet a confirmed etiological link. Future research should prioritize prospective seroepidemiological studies, multicenter pathogen surveillance, and genomic analyses to identify neurovirulent *H. influenzae* strains and host susceptibility factors. Until such data are available, this section should be viewed as a synthesis of emerging evidence and theoretical perspectives rather than a definitive proof of causation.

## 4 Host Factors Influencing GBS Susceptibility

### 4.1 Genetic Predisposition and Immune Regulation

The variability in GBS incidence and severity across populations reflects a strong genetic component that influences host immune responses (Bodis et al. 2018). Several studies have identified HLA class II alleles, particularly HLA‐DQB1 and HLA‐DRB1, as important determinants of susceptibility to infection‐triggered autoimmune neuropathy (Zhao et al. 2020). These alleles modulate antigen presentation and T cell activation, affecting tolerance to ganglioside‐like epitopes derived from microbial antigens. Recent evidence has highlighted that polymorphisms in cytokine regulatory genes, including IL‐17, IL‐10, and TNF‐α, may alter the intensity and duration of inflammatory cascades, influencing disease phenotype and recovery outcomes (Hayat 2021; Sidney et al. 2008; Bouwman and Guchelaar 2024). A 2024 genetic association study further demonstrated that variants in immune synapse‐regulating loci contribute to altered autoantibody production and peripheral nerve vulnerability, reinforcing the concept of a polygenic threshold model in GBS pathogenesis (Iatrou 2018). Collectively, these findings suggest that genetic predisposition shapes immune tolerance thresholds, dictating the likelihood of autoimmune activation following microbial infection.

### 4.2 Microbial Factors and Gene‐Environment Interactions

The likelihood of developing GBS following infection is not solely determined by microbial exposure but also by complex gene‐environment interactions. Certain *C. jejuni* strains express the Cst‐II sialyltransferase gene, which governs LOS sialylation patterns that mimic human gangliosides, such as GM1 and GD1a (Suliman 2024). Individuals carrying specific HLA‐DQB1*0602 and DRB1*1501 haplotypes exhibit stronger cross‐reactive antibody responses to these epitopes, thereby amplifying susceptibility to the axonal variant (AMAN) of GBS (Hayat 2021; Magira et al. 2003). Conversely, infection with H. influenzae or viral pathogens may preferentially trigger immune responses targeting myelin‐associated proteins, resulting in demyelinating (AIDP) forms of the disease. Environmental cofactors, such as antecedent gastrointestinal illness, malnutrition, and regional pathogen exposure, modulate this gene‐pathogen interaction by influencing gut barrier integrity and systemic cytokine profiles. Consequently, host genotype and microbial virulence determine the immunopathological trajectory of GBS.

### 4.3 Immunological Mechanism Linking Susceptibility and Disease Expression

In genetically predisposed individuals, infection‐induced immune activation leads to loss of peripheral tolerance through molecular mimicry and bystander activation. Dysregulated T‐helper (Th17) cell responses and impaired regulatory T‐cell (Treg) function promote sustained inflammation and peripheral nerve injury (Zhang et al. 2024). Elevated levels of IL‐17, IFN‐γ, and TNF‐α have also been reported. Detected in GBS patients during the acute phase, correlating with clinical severity and delayed recovery (Peng et al. 2018). Additionally, autoantibody subclass patterns differ by genotype; patients with HLA‐DQB1 alleles are more likely to produce IgG1/IgG3 antibodies with potent complement‐fixing capacity, exacerbating axonal damage (Schirmer et al. 2016; Jacobs et al. 2008). The interplay between genetic susceptibility, microbial virulence, and immune dysregulation defines not only disease onset, but also clinical heterogeneity. Understanding these host‐pathogen dynamics is critical for developing precision‐based therapeutic interventions, including genotype‐guided immunotherapy and preventive vaccination strategies targeting high‐risk populations.

### 4.4 Infectious Triggers and Clinical Subtype Correlation

Distinct infectious agents are associated with specific GBS subtypes, which reflect the underlying immune mechanisms and antigenic targets. *C. jejuni* infection predominantly induces the AMAN variant through anti‐GM1 and anti‐GD1a antibody responses, leading to axonal degeneration (Latov 2022). Conversely, H. influenzae is more frequently linked to the AIDP subtype, mediated by antibodies against myelin‐associated glycoproteins and Schwann cell epitopes (Ubogu 2024). Viral infections, such as cytomegalovirus and Epstein‐Barr virus, often precipitate mixed demyelinating or sensory variants, highlighting pathogen‐specific immune profiles. These correlations underscore the clinical heterogeneity of GBS and the diagnostic value of serological antibody profiling in determining the disease subtype and prognosis.

## 5 Diagnostic and Analytical Advancements in Guillain–Barré Syndrome

### 5.1 Laboratory Biomarkers and Serological Testing

**Figure 3:**
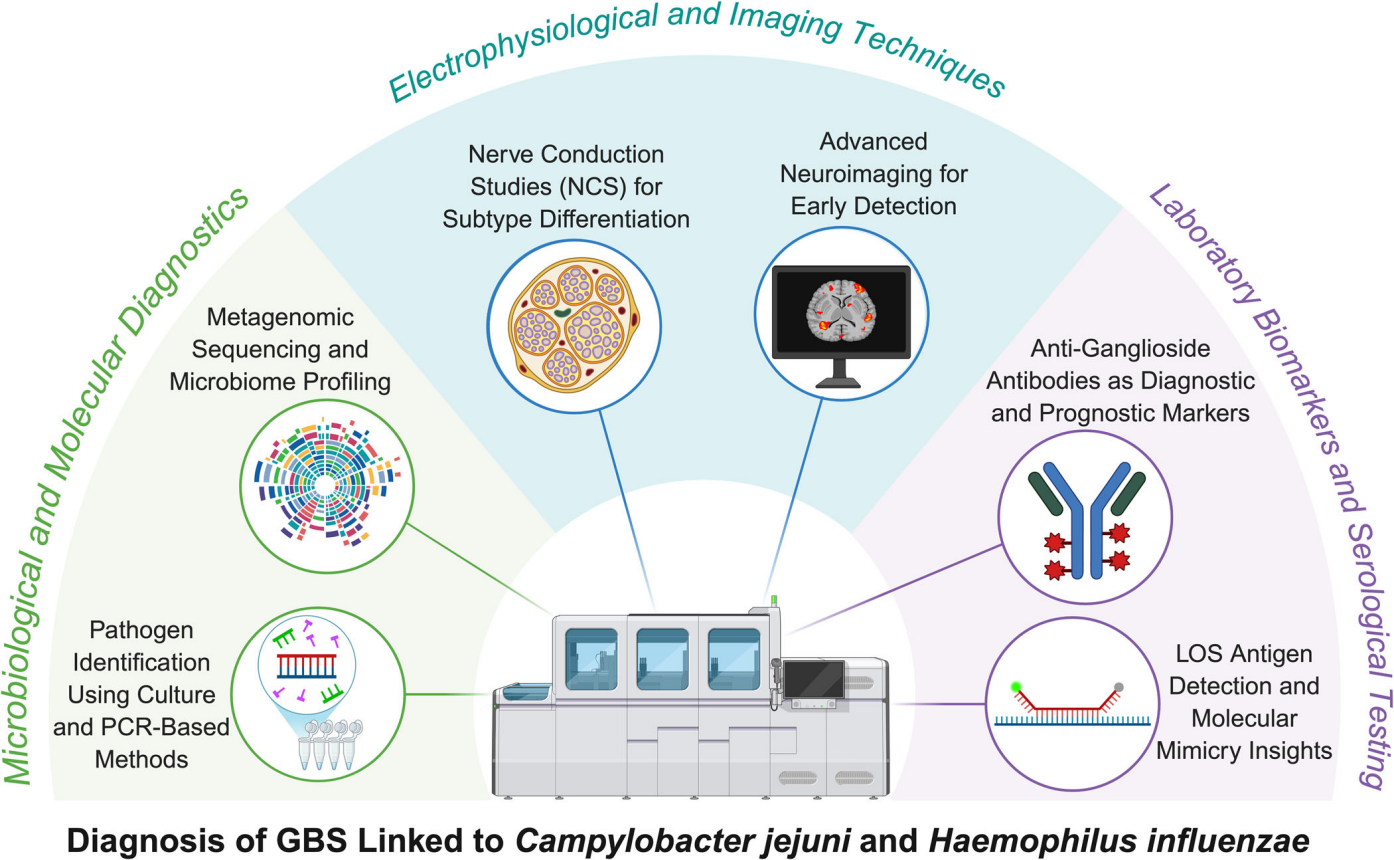
Advancements in the diagnosis of GBS associated with *Campylobacter jejuni* and *Haemophilus influenzae*. Illustrates how various tools, such as microbiology, nerve testing, and antibody detection, are used together to diagnose infection‐related GBS. It emphasizes advances in pathogen identification, nerve damage evaluation, and immune response detection.

Accurate and timely diagnosis of GBS remains a clinical challenge, particularly in differentiating its various subtypes and identifying underlying infectious triggers such as *C. jejuni* and *H. influenzae*. Recent advancements in biomarker discovery, molecular diagnostics, and electrophysiological techniques have significantly improved diagnostic precision (Figure 3).

#### 5.1.1 Anti‐Ganglioside Antibodies as Diagnostic and Prognostic Markers

Autoantibodies targeting neuronal gangliosides are a hallmark of GBS, particularly in cases associated with *C. jejuni*. Serological detection of anti‐GM1, anti‐GD1a, and anti‐GQ1b antibodies significantly enhances diagnostic specificity and correlates with axonal variants, such as AMAN and MFS (Bourque et al. 2015). The IgG subclass of anti‐ganglioside antibodies predominates in *C. jejuni*‐mediated GBS, whereas IgM isotypes are less frequently observed in post‐infectious neuropathies (Malik et al. 2022). An advanced multiplex immunoassay for autoantibody profiling is a high‐throughput serological platform that utilizes microarrays, including bead‐based arrays, which allow the simultaneous detection of multiple autoantibodies, thereby improving both sensitivity and prognostic precision (Sowa et al. 2017).

#### 5.1.2 LOS Antigen Detection and Molecular Mimicry Insights

Because *C. jejuni* and *H. influenzae* possess LOS that mimics neuronal gangliosides, serological assays detecting anti‐LOS antibodies provide valuable evidence of infection‐linked neuropathy. ELISA remains the standard method for identifying LOS‐specific antibodies (Willison and Goodyear 2013). Another commonly used sensor is the Surface Plasmon Resonance (SPR) biosensor, which provides label‐free, real‐time monitoring of LOS‐specific antibody interactions (Butt 2025). Together, these platforms not only aid in confirming the post‐infectious etiology but also deepen the understanding of molecular mimicry and immune dysregulation in GBS.

### 5.2 Microbiological and Molecular Detection Techniques

#### 5.2.1 Pathogen Identification Using Culture and PCR‐Based Methods

Traditional microbiological methods for detecting *C. jejuni* and *H. influenzae* include Selective Culture on Campylobacter‐specific media. Despite its low sensitivity (~ 50%), culture remains the gold standard for confirming *C. jejuni* infection, particularly in resource‐limited settings (Zhang et al. 2003). Second, polymerase chain reaction (PCR) for pathogen‐specific genes, such as *C. jejuni* genes associated with virulence markers, cadF, cdtB, and flaA (Sierra‐Arguello et al. 2021). *H. influenzae* has diagnostic markers for pathogenic strains, such as hpd and ompP6 genes (Price et al. 2015). Another famous technique is Real‐Time Quantitative PCR (qPCR), which offers higher sensitivity than conventional PCR and can quantify bacterial load and differentiate between virulent and nonvirulent strains (Bonnin‐Jusserand et al. 2019).

#### 5.2.2 Metagenomic Sequencing and Microbiome Profiling

Next‐generation sequencing (NGS) technologies have revolutionized pathogen detection, enabling unbiased identification of microbial genomes directly from clinical samples. Whole‐genome sequencing (WGS) facilitates strain typing, virulence gene mapping, and phylogenetic tracking of neurotropic *C. jejuni* and *H. influenzae* isolates (Llarena et al. 2017). Shotgun Metagenomic analyses revealed distinct microbiome alterations in GBS patients, including enrichment of Enterobacteriaceae and depletion of SCFA‐producing commensals (Sajdel‐Sulkowska 2021). CRISPR‐based Diagnostics are emerging as rapid and highly specific platforms for detecting bacterial DNA/RNA signatures linked to GBS pathogenesis (Dubey et al. 2022). These molecular innovations are advancing both diagnostic accuracy and mechanistic insight into host‐microbe interactions.

### 5.3 Electrophysiological and Imaging Techniques

#### 5.3.1 Nerve Conduction Studies for Subtype Differentiation

Electrophysiological assessment remains central to the diagnosis and classification of GBS. AIDP typically shows prolonged conduction velocities and conduction blocks, whereas acute motor‐sensory axonal neuropathy (AMAN/AMSAN) variants present with markedly reduced compound muscle action potentials but preserved conduction velocities (Khadilkar et al. 2024). The incorporation of machine learning into nerve‐conduction data analysis enhances diagnostic precision and differentiates GBS from overlapping neuropathies (Alganmi 2024).

#### 5.3.2 Advanced Neuroimaging for Early Detection

Although historically underused, magnetic resonance neurography (MRN) and diffusion‐weighted imaging (DWI) are gaining value in early GBS detection, revealing cauda equina enhancement and subtle brainstem white matter changes before clinical deterioration (Madhavan et al. 2020; Ellingson et al. 2014). PET imaging targeting neuroinflammatory markers such as translocator protein (TSPO) tracers enables visualization of microglial activation as an early marker of neuroinflammation (Corica et al. 2023). The integration of multi‐omics biomarker discovery, which combines genomics, proteomics, and metabolomics, promises to identify new diagnostic and prognostic signatures. Parallel advances in AI driver predictive models and nanomaterial‐based biosensors have paved the way for rapid point‐of‐care GBS diagnostics (Guo et al. 2024). Collectively, these technological innovations redefine early detection, enabling timely therapeutic intervention and individualized patient management.

## 6 Therapeutic Interventions and Emerging Strategies

### 6.1 Established Immunomodulatory Therapies

The current mainstay of GBS management is immunomodulation and intensive supportive care. Early initiation of either intravenous immunoglobulin (IVIg) or plasma exchange (PLEX) within the first 2 weeks of symptom onset remains the cornerstone of therapy, both demonstrating equivalent efficacy in hastening recovery and reducing ventilatory dependency (Katsumoto et al. 2022). IVIg neutralizes pathogenic antibodies, inhibits complement activation, and suppresses Fc receptor‐mediated immune responses (Bournazos et al. 2020). In contrast, PLEX directly removes circulating autoantibodies and inflammatory mediators, thereby reducing immune attacks on the peripheral nerves. Combination therapy does not confer any additional benefits and is generally not recommended. Corticosteroids, though previously considered, have shown no significant improvement in outcomes and may even delay recovery in some patients (Tiwana et al. 2005). Consequently, their use is limited to specific scenarios such as refractory inflammation or overlapping autoimmune syndromes. Supportive measures, including close monitoring of respiratory function, prevention of deep vein thrombosis, and multidisciplinary rehabilitation, are crucial for functional recovery and long‐term quality of life (Ruan et al. 2023).

### 6.2 Targeted Immunotherapy and Cytokine Modulation

Recent advances in neuroimmunology have prompted the exploration of targeted therapies that modulate the specific immune pathways implicated in GBS pathogenesis. Monoclonal antibodies against complement component C5 (e.g., eculizumab) have shown promising results in reducing nerve injury by blocking the activation of terminal components (Giorgio et al. 2021). Similarly, anti‐IL‐6 receptor therapy (Tocilizumab) and anti‐IL‐17 monoclonal antibodies (Secukinumab) are being investigated for their potential to mitigate cytokine‐mediated neuroinflammation (Milovanovic et al. 2020). Emerging data also indicate a role for neonatal Fc receptor (FcRn) inhibitors, which accelerate the catabolism of pathogenic IgG antibodies, thereby shortening disease duration (Nelke et al. 2022). These precision immunotherapies represent a paradigm shift from generalized immune suppression to the mechanism‐based modulation of disease‐specific pathways. However, the cost, limited accessibility, and small clinical trial size currently constrain their widespread implementation.

### 6.3 Microbiome Modulation and Gut‐Nerve Axis Therapeutics

An emerging area of therapeutic interest is the gut‐nerve axis, wherein the intestinal microbial composition influences systemic immune responses relevant to GBS. Studies have demonstrated that post‐infectious dysbiosis, especially following *C. jejuni* enteritis, alters SCFA (short‐chain fatty acid) profiles and weakens mucosal immune tolerance (Jalanka et al. 2023). Experimental restoration of gut eubiosis using probiotics, prebiotics, or synbiotic formulations has been shown to downregulate Th17‐mediated inflammation and promote regulatory T‐cell induction, potentially mitigating disease severity (Tanabe 2013). Thus, microbiome‐directed interventions could complement immunotherapy by reducing systemic immune activation and promoting neural repair. Future studies should evaluate systemic immune activation and the promotion of neural repair. Future studies should evaluate standardized probiotic strains, dose regimens, and timing relative to the acute and recovery phases of GBS to establish its translational feasibility.

### 6.4 Vaccine Development and Preventive Immunology

Given the established link between certain infectious agents and GBS, vaccine development represents both an opportunity and a challenge, and efforts are ongoing to create LOS‐based conjugate and subunit vaccines that provide protection without inducing autoimmune mimicry (Poly et al. 2019). Similarly, conjugate and subunit vaccines provide protection without inducing an autoimmune mimicry. Similarly, conjugate vaccines against *H. influenzae* type b (Hib) have drastically reduced invasive infections and extended the potential for post‐infectious neurological complications (Olivieri et al. 2021). To minimize autoimmune risk, modern vaccine designs increasingly employ epitope engineering to eliminate ganglioside‐mimicking structures. Integrating pathogen genomic surveillance with host immunogenetic profiling could further refine vaccine safety and efficacy (Bentley and Lo 2021). Personalized immunoprophylaxis may ultimately prevent infection‐induced GBS in genetically or immunologically susceptible individuals.

### 6.5 Integrative and Future Therapeutic Directions

An evolving understanding of GBS pathogenesis highlights the need for multidimensional therapeutic strategies that bridge microbiology, immunology, and systems biology. Future directions include the application of systems immunology, transcriptomic profiling, and AI‐based biomarker discovery to predict the disease course and treatment responsiveness (Chang et al. 2025). Personalized therapeutic frameworks that integrate genetic risk assessment, microbiome composition, and immune profiling could enable precision care tailored to individual patient signatures. Ultimately, bridging basic microbial research with clinical practice will be essential for redefining GBS management from reactive interventions to proactive prevention.

## 7 Bridging the Translational Gap: Clinical Trials and Epidemiological Silence in GBS Pathogen Surveillance

As of the current review of global clinical trial databases, notably ClinicalTrials.gov, the translational research landscape examining the microbial triggers of GBS remains surprisingly underdeveloped (Table 1). To date, only one completed clinical trial (NCT02493725) has been officially recorded that directly explores the role of *C. jejuni* in GBS pathogenesis. This observational study aimed to investigate immune responses triggered by *C. jejuni* infection and their correlation with the onset of GBS. Although the trial summary lacks granular data on its cohort structure and outcome measures, its existence confirms the scientific community's recognition of the need to explore infectious etiologies in GBS. Importantly, this study provides preliminary immunopathological evidence supporting the role of *C. jejuni* lipo‐oligosaccharides in molecular mimicry, reinforcing the theory of ganglioside autoantibody‐mediated axonal damage.

**Table 1:** Clinical trial landscape investigating bacterial etiologies in GBS.

Pathogen	Clinical trial	Title	Key findings
*Campylobacter jejuni*	NCT02493725 – Completed	Role of *C. jejuni* infection in the pathogenesis of GBS.	The first and only recorded trial investigating *C. jejuni*‐induced GBS suggests a role for molecular mimicry and immune triggers.
*Haemophilus influenzae*	Not available	No clinical trials registered as of April 2025.	Despite its clinical relevance, *H. influenzae* remains understudied, and epidemiological data suggests an under‐recognized involvement.

In contrast, no registered clinical trial data are available on *H. influenzae* as a causative agent of GBS. However, the absence of trial documentation does not equate to epidemiologically irrelevant data. *H. influenzae* has been repeatedly implicated in sporadic clinical reports and retrospective case series, often preceding atypical GBS presentations, particularly in pediatric and immunocompromised cohorts. Its presence in the upper respiratory tract and known neurotropic potential through hematogenous spread suggest an underappreciated role in neuropathogenic immune activation. The lack of structured investigations into *H. influenzae*‐associated GBS represents a critical gap in pathogen surveillance and clinical microbiology. Given the increasing interest in post‐infectious neurological syndromes, especially in the wake of viral pandemics and antibiotic resistance, future clinical trials must be strategically designed to include *H. influenzae* serotypes as variables of interest. Multicenter surveillance efforts, longitudinal immune profiling, and prospective seroepidemiological studies are urgently needed to validate the role in GBS onset and progression. While *C. jejuni* remains the best‐studied microbial antecedent of GBS within clinical trial frameworks, the current scarcity of data does not reflect the full spectrum of microbial contributors to GBS. This diagnostic and investigational vacuum calls for a paradigm shift in how we conceptualize infection‐induced autoimmunity, urging researchers to broaden the scope of microbial candidates and translate clinical suspicions into empirical data.

## 8 Therapeutic Strategies and Future Perspectives

### 8.1 Current Treatment Approaches

The therapeutic management of GBS, particularly in cases triggered by *C. jejuni* or *H. influenzae*, remains anchored in immunomodulation and supportive care (Table 2). Currently, two frontline immunotherapeutic modalities, intravenous immunoglobulin (IVIG) and plasma exchange (PLEX), constitute the cornerstone of GBS treatment (Zaki et al. 2023). These interventions have been empirically established to mitigate aberrant autoantibody‐mediated nerve injury by neutralizing pathogenic immunoglobulins (IVIG) or physically removing them from circulation via PLEX (Jacob et al. 2021). Despite their widespread use, heterogeneity in patient responsiveness remains a significant therapeutic dilemma. For instance, IVIG resistance has been increasingly reported in patients with high anti‐ganglioside antibody titers, particularly anti‐GM1 and GD1a IgG isotypes, which are common in *C. jejuni*‐associated AMAN subtypes (Matà et al. 2006). This underscores the pressing need for stratified medicine approaches and predictive biomarkers of therapeutic efficacy. Supportive measures are vital for preventing secondary complications. These include mechanical ventilation for patients with bulbar or respiratory involvement, neuropathic pain control using gabapentinoids or tricyclics, thromboprophylaxis, and neurorehabilitation. Recent literature emphasizes that early multidisciplinary rehabilitation can accelerate functional recovery and enhance the long‐term quality of life, particularly in patients with axonal involvement (Prada et al. 2020).

**Table 2:** Advantages and disadvantages of the therapeutic management used in GBS.

Therapeutic strategy	Advantages	Disadvantages
Intravenous Immunoglobulin (IVIG)	▪ Easy to administer ▪ Effective in early GBS ▪ Less invasive than PLEX	▪ Expensive ▪ Not effective in all patients (e.g., anti‐GM1+subtypes) ▪ Potential IVIG resistance
Plasma exchange	▪ Directly removes pathogenic antibodies ▪ Proven efficacy	▪ Invasive and resource‐intensive ▪ Requires hospital infrastructure
Supportive care	▪ Critical for preventing complications ▪ Includes pain control, ventilation, rehab	▪ Does not address underlying immune cause ▪ Highly individualized needs
Monoclonal antibodies	▪ Targeted action against specific immune components ▪ Potential to reduce immune off‐target effects	▪ Experimental phase ▪ Not yet widely available clinically
Probiotics/Microbiome modulation	▪ Potential to reduce immune dysregulation ▪ Noninvasive approach	▪ Lacks standardized protocols ▪ Clinical efficacy under investigation
Toll‐like receptors (TLR)	▪ Targets upstream immune activation ▪ Theoretical prevention of molecular mimicry	▪ Mostly preclinical ▪ Safety and specificity yet to be confirmed
Faecal microbiota transplantation	▪ Can restore immune balance via gut‐brain axis	▪ Safety standardization and long‐term effects unclear
Gene editing (CRISPR/Cas9)	▪ Potential to precisely disrupt autoimmunity pathways	▪ Still in research stage ▪ Ethical and technical challenges remain
Vaccination (Subunit/LOS‐deficient)	▪ Theoretical protection from trigger organisms without autoimmunity	▪ Development is challenging ▪ Risk of molecular mimicry must be minimized

### 8.2 Targeted Therapies and Emerging Approaches

Given the autoimmune etiology of GBS, precision‐targeted immunotherapies have emerged as promising frontiers. Experimental models and preliminary clinical investigations have evaluated the potential of anti‐ganglioside monoclonal antibodies to selectively inhibit autoantibody binding to peripheral nerves, potentially preserving axonal integrity without inducing global immunosuppression (Goodfellow and Willison 2018). Another paradigm‐shifting concept is the manipulation of host microbiota to recalibrate immune tolerance. Gut microbiome studies have demonstrated significant dysbiosis in GBS patients after C. *jejuni* infection, characterized by increased pro‐inflammatory taxa and reduced butyrate producers (Ahmed 2022). Therapeutic strategies, including probiotic supplementation with *Bifidobacterium* and *Faecalibacterium* and fecal microbiota transplantation (FMT), are being explored to attenuate molecular mimicry‐induced immune activation (Zhang et al. 2023). Concurrently, interest is growing in TLR antagonists, particularly TLR4 inhibitors, given their central role in recognizing *C. jejuni* LOS and priming innate immune responses. Targeting these upstream sensors could disrupt the feed‐forward loop of inflammation and autoreactivity that underlies the pathogenesis of GBS. Moreover, gene‐editing technologies, such as CRISPR/Cas9, are under exploratory research for silencing genes involved in autoimmune signaling cascades, although this remains in the realm of preclinical investigation.

### 8.3 Vaccination Strategies and Prevention

Although no vaccines are available for GBS prevention, this topic remains contentious and critical. Molecular mimicry, in which bacterial LOS or viral proteins mimic human gangliosides, suggests the theoretical possibility of designing vaccines that block pathogen invasion without triggering autoimmunity (Suliman 2024). However, vaccines have occasionally been implicated as triggers for GBS, particularly influenza and COVID‐19. Epidemiological studies have revealed a modestly increased incidence of GBS following certain viral vaccines, particularly in patients with a history of GBS. Thus, postvaccination surveillance and personalized risk‐benefit assessments are essential components of GBS preventive strategies (Montero 2024). In the future, subunit vaccines or LOS‐deficient bacterial mutants could offer safer immunogenic platforms devoid of neuropathogenic mimicry.

## 9 Future Directions

Ongoing research into the microbial and immunological aspects of Guillain–Barré syndrome (GBS) continues to expand our understanding of this complex neuroimmune disorder. Future studies should focus on integrating multi‐omics data, including genomics, transcriptomics, and metabolomics, to uncover host‐pathogen interactions that predispose individuals to post‐infectious autoimmunity. Large‐scale genome‐wide association studies (GWAS) and immunoprofiling analyses will be instrumental in defining genetic susceptibility and identifying molecular biomarkers predictive of disease onset and recovery The gut‐nerve axis represents a promising yet underexplored therapeutic target. Future studies should investigate how restoring microbial homeostasis through probiotics, prebiotics, or postbiotics can modulate systemic immune activation and promote neural repair. Additionally, precision immunotherapies, such as monoclonal antibodies against complement or cytokine pathways, warrant further evaluation in controlled clinical trials to optimize their safety and efficacy.

From a preventive standpoint, advances in pathogen genomics and vaccine design offer opportunities to mitigate infection‐related GBS risks. Rational design of *Campylobacter jejuni* vaccines that exclude ganglioside‐mimicking epitopes, coupled with enhanced surveillance of *Haemophilus influenzae* strains, may enable safer immunization strategies for at‐risk populations. Finally, artificial intelligence (AI)‐driven predictive models incorporating clinical, serological, and microbiome data may revolutionize early diagnosis and enable personalized therapeutic interventions for GBS.

## 10 Conclusion

GBS exemplifies a complex interplay between microbial infection, host genetic predisposition, and immune dysregulation, which culminates in acute peripheral neuropathy. The most frequently implicated pathogens, *C. jejuni* and *H. influenzae*, initiate autoimmune responses through molecular mimicry in which bacterial LOS structures resemble host gangliosides. This mimicry triggers the production of cross‐reactive antibodies that target neuronal membranes, leading to axonal or demyelinating injuries. The expression of virulence genes, such as Cst‐II, in *C. jejuni* further enhances pathogenicity, particularly in individuals harboring susceptible HLA alleles that amplify antibody‐mediated complement activation.

Although GBS remains a rare complication of infection, it carries substantial morbidity owing to its unpredictable course and potential for respiratory failure or long‐term neurological sequelae. Early recognition and prompt initiation of immunotherapy (IVIg or PLEX) remains critical for improving recovery outcomes. The emergence of targeted biological therapies, including complement inhibitors, anti‐cytokine monoclonal agents, and FcRn blockers, offers promising avenues for precision‐based management. Simultaneously, advances in microbiome modulation, including probiotic and synbiotic formulations, highlight a novel therapeutic frontier that leverages gut‐immune interactions to restore immune tolerance. Preventive efforts should focus on vaccines and pathogen surveillance. The rational design of *C. jejuni* vaccines that avoid ganglioside‐mimicking epitopes, combined with ongoing *H. influenzae* immunization programs, may reduce the infection‐driven GBS incidence in high‐risk populations.

In summary, GBS represents a paradigm of post‐infectious autoimmunity in which microbiological and immunogenetic insights converge. The path forward lies in interdisciplinary collaboration that bridges microbial genomics, clinical neurology, and immunotherapy to transform the understanding, prevention, and treatment of this debilitating neuroimmune disorder. This review emphasizes that integrating microbiological insights, systems immunology, and precision medicine is crucial in redefining both the prevention and management of GBS.

## Author Contributions

**Aswathi Ramesh:** data curation (equal), formal analysis (equal), resources (equal), validation (equal), visualization (equal), writing – original draft (equal). **Rajasekaran Subbarayan:** conceptualization (equal), data curation (equal), formal analysis (equal), project administration (equal), resources (equal), supervision (equal), validation (equal), visualization (equal), writing – original draft (equal). **Dhasarathdev Srinivasan:** formal analysis (equal), validation (equal), writing – review and editing (equal). **Ranjith Balakrishnan:** formal analysis (equal), validation (equal), writing – review and editing (equal). **Rupendra Shrestha:** conceptualization (equal), data curation (equal), formal analysis (equal), project administration (equal), resources (equal), supervision (equal), validation (equal), visualization (equal), writing – original draft (equal). **Ankush Chauhan:** formal analysis (equal), validation (equal), writing – review and editing (equal).

## Ethics Statement

The authors have nothing to report.

## Consent

The authors have nothing to report.

## Conflicts of Interest

The authors declare no conflicts of interest.

## Data Availability Statement

The authors have nothing to report.
